# Changes in chemical and ultrastructural composition of ameroid constrictors following *in vitro* expansion

**DOI:** 10.1371/journal.pone.0207471

**Published:** 2018-11-15

**Authors:** Thomas S. Anderson, Graham A. Rance, Long Jiang, Matthew J. Piggott, Elinor J. Field, Guillaume P. Chanoit

**Affiliations:** 1 Bristol Veterinary School, Faculty of Health Sciences, University of Bristol, Langford, Bristol, United Kingdom; 2 Nanoscale and Microscale Research Centre, Faculty of Science, University of Nottingham, Nottingham, United Kingdom; 3 Advanced Materials and Healthcare Technologies, School of Pharmacy, Faculty of Science, University of Nottingham, Nottingham, United Kingdom; University of Lincoln, UNITED KINGDOM

## Abstract

**Objective:**

To (1) characterise the chemical and ultra-structural composition of ameroid constrictors, at a native state and during in vitro expansion and (2) determine the presence of irritant compounds at the surface or within the bulk of the constrictor.

**Methods:**

Twelve sterile, commercially packaged ameroid constrictors (3 repeats of 3.5 mm, 5 mm, 6 mm and 7 mm internal diameter) were analysed by time-of-flight secondary ion mass spectrometry, Raman spectroscopy, attenuated total reflectance Fourier transform infrared spectroscopy and scanning electron microscopy.

**Results:**

Ameroid constrictors have a composition commensurate with casein with little-to-no intra- or inter- constrictor variation. Microscopic analysis indicated that the topographical features of the constrictor surfaces were consistent between all constrictors. Following *in vitro* expansion there was a reproducible decrease in Ca^+^ ion content, little-to-no variation in secondary protein structure and morphological changes including the presence of surface aggregates present only at the inner surface of the ameroid constrictor. The potential irritant polydimethylsiloxane was found on the constrictor surface. A trace quantity of an ion fragment assigned as formaldehyde was detected; however, the extremely low level is thought highly unlikely to play a role as an inflammatory trigger clinically.

**Discussion:**

There is a high degree of inter- and intra-constrictor homogeneity from different batches, and reproducible ultrastructural changes following *in vitro* expansion. Variations occur in both the surface chemistry and topography of the device during closure, which can potentially affect the biomaterial-host interface. Ameroid constrictor closure mechanism is likely involving calcium-mediated inter-protein interactions rather than the imbibition of water only.

## Introduction

The ameroid ring constrictor (ARC) is a device that has historically been used in research to create *in vivo* models of gradual arterial/venous occlusion, in order to replicate chronic ischaemic disease. [1–3] In Veterinary medicine, it is almost exclusively known to be used clinically to obtain gradual occlusion of single congenital portosystemic shunts in small animals. [4–6] An ARC’s manufacturing process involves combining rennet casein, a by-product of cheese manufacture, with water. Formaldehyde is added during the process to harden the casein. Finally, a stainless steel ring is placed around the ARC to complete the construct. [2]

The hygroscopic nature of an ARC allows it to swell centripetally to provide external compression upon the vessel it surrounds, reducing luminal diameter. However, *in vivo* studies following ARC placements around peripheral veins have shown that flow through the shunt is usually halted before expected due to external compression alone, and the same studies have shown that perivascular inflammation (characterised by neutrophilic, histiocytic and lymphocytic infiltrates) and fibrosis, as well as intravascular thrombosis provide a significant contribution to attenuation of the vessel lumen. [7–10]

Little is known about the mechanisms that underpin this inflammatory reaction and the formation of an intravascular thrombus. It is hypothesised that the response could be induced directly by the ameroid, or by any residual formaldehyde on the surface or subsurface of the ARC. However, the presence and amount of formaldehyde present in ARCs commercially available for use in portosystemic shunt surgery is not known. [7–9]

Another hypothesis is that expansion of the ARC could either expose irritant compounds or induce modifications in the ultrastructure of the protein network within the ARC, making the latter more “thrombogenic”.

Recent *in vitro* studies have noted that the ionised calcium concentration of the perfusate surrounding the ARC increases significantly during the course of the *in vitro* expansion. [11] This is thought to be as a result of Ca^2+^ ions leaching from the ARC, and is hypothesised to be a potential trigger source for thrombosis, due to the integral role of calcium in several steps of the coagulation cascade. [11] However, it is unknown how calcium leaching influences the ultrastructure of the ARC during expansion.

A key factor identified in the rate of ARC closure is the protein concentration of the medium in which it expands and canine plasma perfusate has been used to model the *in vivo* environment. [9,11,12] It is not clear though how exposure to canine plasma influences the ultrastructure and chemical composition of the ARC. In addition to this, recent studies have identified stark variations in the chemical and physical properties of thin-plastic films used for attenuation of portosystemic shunts, [13] but it is unknown whether ARCs used in the veterinary clinical setting have ultrastructural and chemical homogeneity between different batches.

The goals of our study were therefore to (1) describe the chemical and ultrastructural composition of ARCs in their native state, following *in vitro* expansion using sterile commercially packed ARCs from different batches, and (2) determine the presence of irritant compounds at the surface or within the bulk of the ARC.

Our hypotheses were that: (1) ARCs were chemically consistent with casein, with a high degree of intra- and inter-constrictor homogeneity between batches; (2) the chemical and ultrastructural composition showed reproducible alteration following immersion in canine plasma; and (3) irritant compounds were not present at the surface or in the bulk of the sterile ARC.

## Materials and methods

Twelve standard ARCs, each of a different internal diameter (three each of 3.5, 5, 6 and 7 mm) were obtained sterile in packaging direct from the manufacturer. Different sized constrictors were chosen to ensure they were from different batches. Under surgical conditions of sterility, the ARCs were removed from their packaging and processed as follows to allow chemical characterisation. The stainless steel outer ring and key was removed from each constrictor, and the ARC sectioned in half across its short axis using a small surgical hack saw. Four methods of chemical analysis were used on all constrictors, namely time-of-flight secondary ion mass spectrometry (ToF-SIMS) permitting surface-sensitive (top ~ 3 nm) chemical analysis, Raman and attenuated total reflectance Fourier transform infrared (ATR-FTIR) spectroscopies to probe the chemical composition of the subsurface to bulk, and scanning electron microscopy (SEM), allowing characterisation of the surface topography. One half of each constrictor was analysed in the native state (hereafter denoted as “Native”) and one half of each constrictor after 6 weeks of plasma immersion (hereafter denoted as “Post-Immersion”).

### ToF-SIMS

Samples were analysed with a mass spectrometer (ToF-SIMS IV, ION-TOF GmbH, Germany) using a bismuth liquid metal primary ion gun (Bi^3+^, 25kV) for analysis and an argon gas cluster (Ar_n_^+^, 1.5kV) sputter source for depth profiling. As the samples were not conductive, charge compensation was applied in the form of a low energy (<20 eV) electron floodgun and analysis was maintained in the static regime with a primary ion dose density of < 1. Depth profiling was performed over an area of 400 × 400 μm with analysis of the central 200 × 200 μm area at 256 × 256 pixel resolution. Depth profiling was performed in ‘non-interlaced’ mode with 1 frame of analysis followed by 5 seconds of sputter time. Data acquisition and analysis were performed using the software recommended for the mass spectrometer used (SurfacelLab 6, ION-TOF GmbH, Germany).

### Raman spectroscopy

Raman spectroscopy was performed using a spectrometer equipped with an automated *xyz* stage (Horiba Jobin Yvon LabRAM HR Raman spectrometer, Japan). Spectra were acquired in the range 900–3200 cm^-1^ using a 785 nm laser at 12 mW power, a 50× objective and a 300 μm confocal pinhole. The spatial resolution in this configuration is ~1 and ~30 μm in the lateral (xy) and axial (z) dimensions respectively. To simultaneously scan a range of Raman shifts, a 600 lines mm^-1^ rotatable diffraction grating along a path length of 800 mm was employed. Spectra were detected using a Synapse CCD detector (1024 pixels) thermoelectrically cooled to −60°C. Before spectra collection, the instrument was calibrated using the zero order line and a standard Si(100) reference band at 520.7 cm^-1^. In a typical measurement, the ARCs were irradiated for 10 minutes to reduce the background fluorescence by photobleaching, then spectra were collected with an acquisition time of 30 seconds and 16 accumulations to improve the signal to noise ratio. Spectra were baseline corrected for residual fluorescence using a third-order polynomial fitting model. Peak deconvolution in the amide I region 1590–1720 cm^-1^ was performed by fitting eight Gaussian bands using Labspec 6.4.3 software.

### ATR-FTIR

Spectra were recorded using a spectrophotometer with an diamond ATR (attenuated total reflectance) module (Agilent Cary 630 FTIR Spectrophotometer with diamond ATR module, Agilent Technologies, Cal, USA). Background spectra were obtained before the sample was loaded onto the crystal, and analysis with a spectral resolution of 2 cm^-1^ in the range 650–4000 cm^-1^ was undertaken. Spectra were processed using a commercial software package.

### SEM

Electron micrographs were taken using an environmental scanning electron microscope (Quanta 650 ESEM, Thermo Fisher Scientific, Massachusetts, USA). Samples were mounted on aluminium stubs and fixed using carbon cement. Low vacuum operation of the SEM allowed the constrictors to be examined without any conductive coatings. A working pressure of 60 Pa was chosen, and an accelerating voltage of 10 kV. Working distance was around 10 mm. All imaging was carried out in secondary electron (SE) mode. Magnification was varied as appropriate to assess visible structure and is documented in each image.

### Plasma immersion

One half of each constrictor was placed into an individual well containing 10 mL of canine plasma. In order to minimise bacterial and fungal contamination the plasma was mixed with Penicillin-Streptomycin-Amphotericin B solution to achieve a concentration of streptomycin (100 μg mL^-1^), penicillin (100 U mL^-1^) and amphotericin B (0.25 μg mL^-1^), and replaced completely every 14 days. The samples were incubated for six weeks at 38 ^o^C and 5% CO2. Prior to analysis the samples were washed with sterile saline.

## Results

### Native state analysis

**Fig 1:**
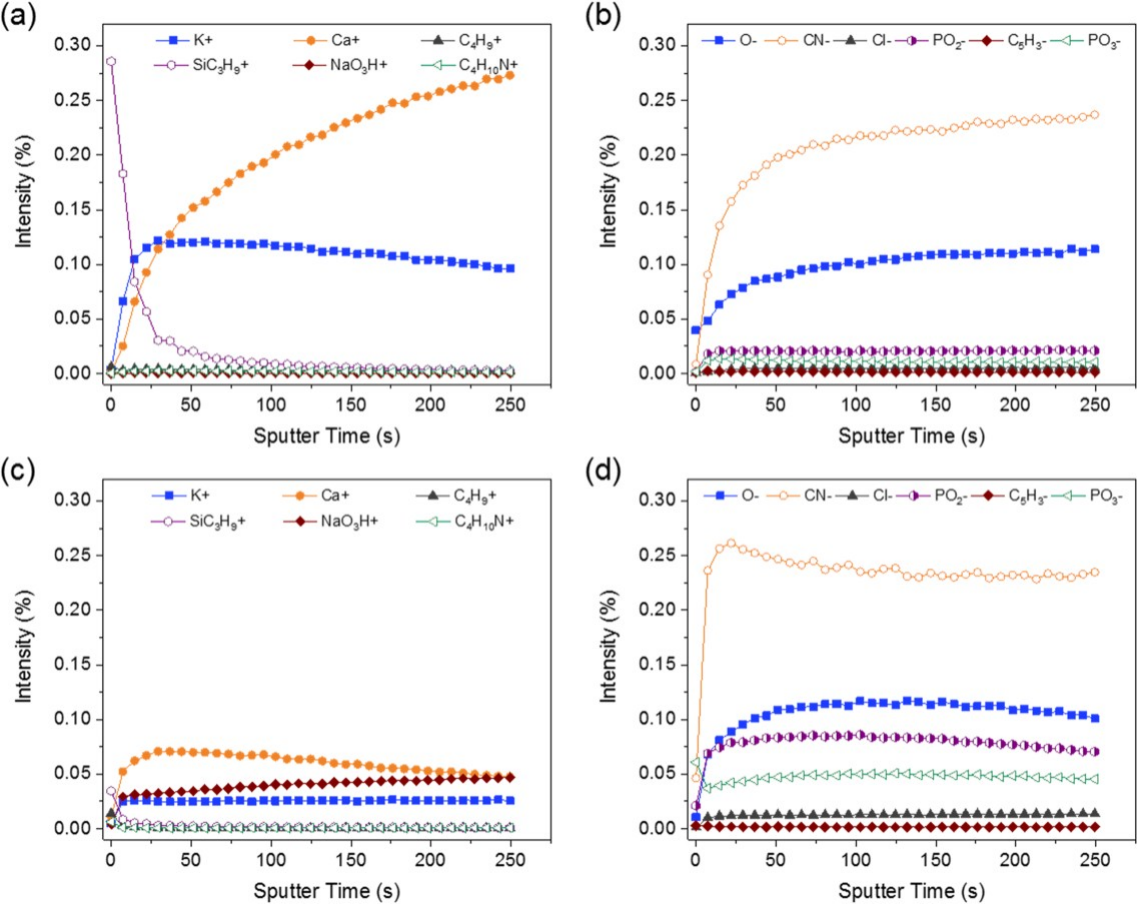
ToF-SIMS depth analysis: (a) positive and (b) negative polarity profiles of the native 5 mm ARC; (c) positive and (d) negative polarity profiles of the 5 mm ARC subsequent to exposure to canine plasma. It is important to note that the dominant ion in the positive polarity profile of all the ARCs was Na^+^ (*m*/*z* = 22.99). However, this has been deliberately excluded from the corresponding profiles to allow visualisation of the other important mass fragments, which would be scaled to suppression if included. The variation of Na^+^ intensity with depth was seen to be directly analogous to the trend observed for NaO_3_H^+^, and this has therefore been used as a surrogate.

The surface structure of the native ARCs as identified by ToF-SIMS was dominated by ions corresponding to K^+^ (*m*/*z* = 38.96), Ca^+^ (*m*/*z* = 39.95), O^-^ (*m*/*z* = 15.99), CN^-^ (*m*/*z* = 26.00) and Cl^-^ (*m*/*z* = 34.97), with ions of lower intensity corresponding to the phosphate groups PO_2_^-^ (*m*/*z* = 62.96) and PO_3_^-^ (*m*/*z* = 79.96) (Fig 1). This was commensurate with that expected for a biomaterial such as the phosphoprotein casein.

The ToF-SIMS depth profiles indicated that the intensity of the mass fragment associated with SiC_3_H_9_^+^ (*m*/*z* = 73.05), despite an initial spike at the top surface, dropped dramatically after a short duration of sputtering. An analogous trend was observed for C_4_H_9_^+^ ions (*m*/*z* = 57.06) indicative of an enrichment of these two species at the surface of the constrictor relative to the bulk. Conversely, the intensity of the ions such as Ca^+^, K^+^, Cl^-^ and CN^-^, increases correspondingly with increasing sputtering time suggesting an association with bulk chemistry. The SiC_3_H_9_^+^ ions have been attributed to polydimethylsiloxane (PDMS), an organic contaminant potentially introduced during handling and/or exposure to atmosphere.

**Fig 2:**
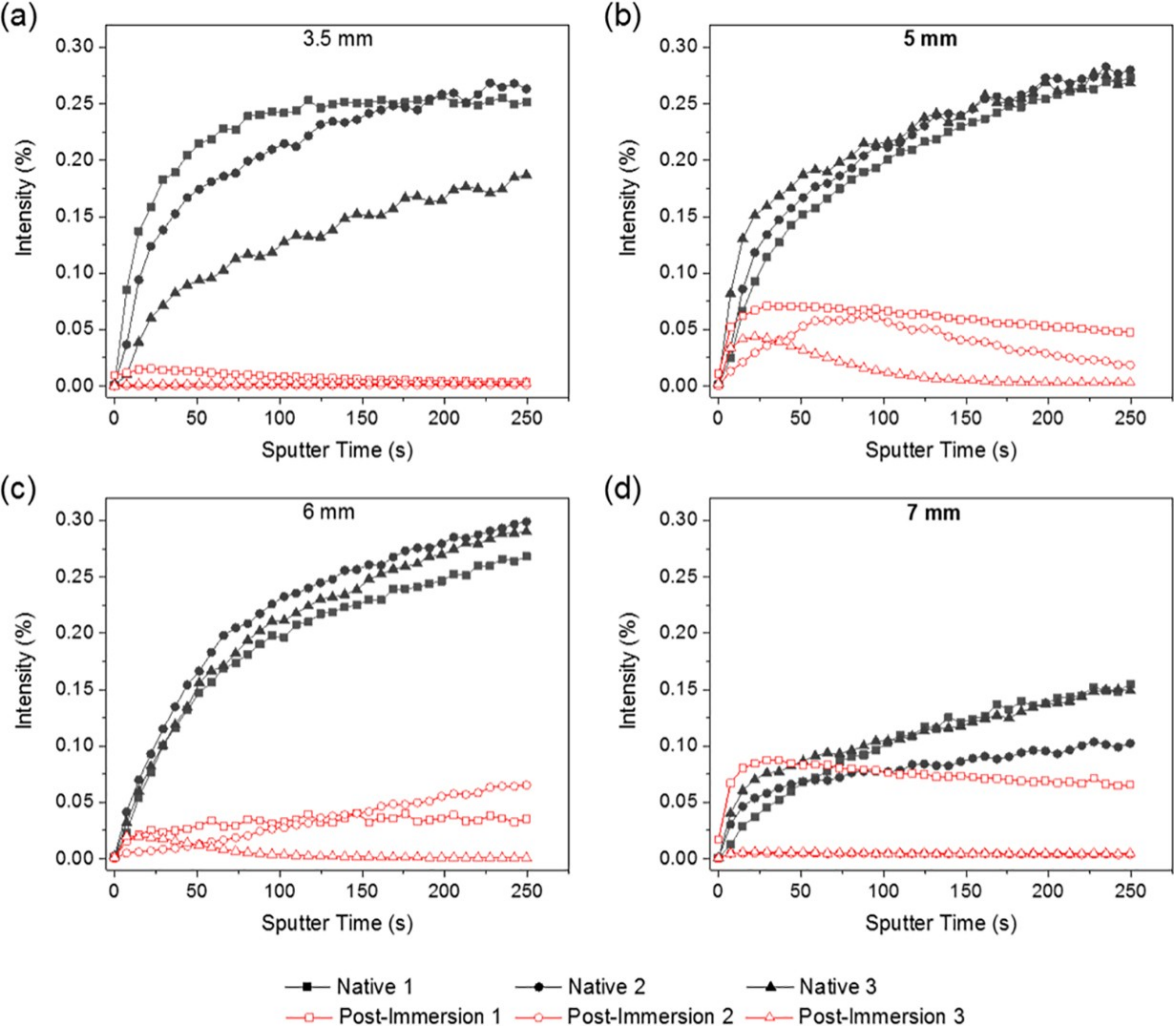
Ca^+^ profiles for ARCs: (a) 3.5 mm, (b) 5 mm, (c) 6 mm and (d) 7 mm. For all constrictors in the native state calcium was a dominant cation associated with the bulk of the constrictor. Following plasma immersion there is an overall depletion from the bulk from all constrictors with an enrichment at the subsurface, indicating movement from the bulk to the superficial layers.

Analysis of three separate constrictors of each of the four sizes (Fig 2) indicated that Ca^+^ was present in all ARCs, increasing in intensity with increasing sputtering time, with only subtle intra-constrictor heterogeneity observed.

The CH_3_O^+^ ion (m/z = 31.03), nominally attributed to formaldehyde was detected consistently in the ARCs (S1 Fig), but with a low intensity not substantially above the detection limit of the instrument (background noise) (S2 Fig). Due to the fragmentation process inherent to ToF-SIMS, other larger organic species may generate this ion during analysis, and as such there is no conclusive evidence formaldehyde is present at the sample top surface. However, it cannot be explicitly ruled out that trace concentrations could be present.

**Fig 3:**
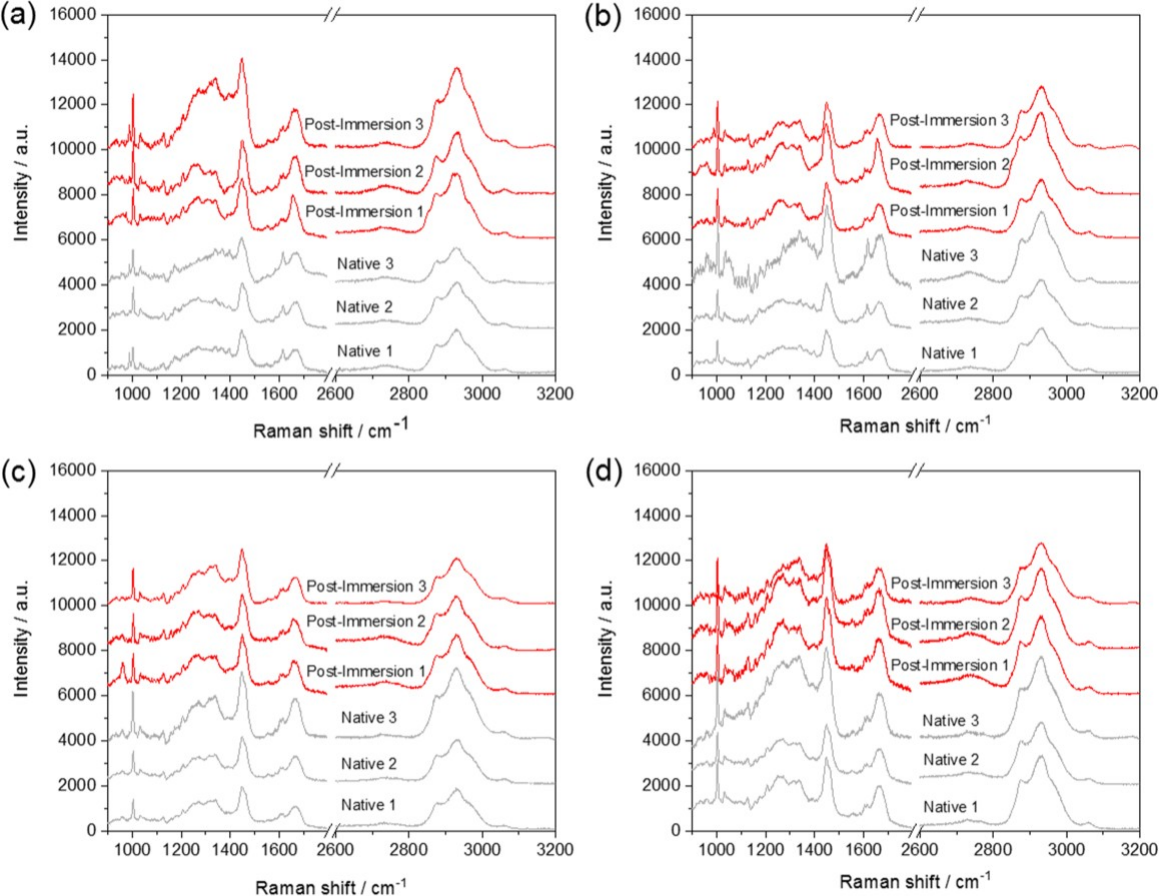
Raman spectra of the different-sized ARCs (a) 3.5 mm, (b) 5 mm, (c) 6 mm and (d) 7 mm, measured in triplicate to evaluate the homogeneity of the constrictors, in the native state and post-immersion in canine plasma. Spectra have been shifted on the vertical axis for clarity.

The Raman spectra of all of the ARCs (Fig 3) were analogous to those expected for globular proteins, with a number of bands noted and the significant ones tentatively assigned. There were very little differences in the Raman spectra collected from different areas within the same ARC or indeed different ARCs of the same diameter or from ARCs of different diameters. Furthermore, deconvolution of the amide I band at ~1650 cm^-1^, used to describe the secondary protein structure including the ratio of α-helix to β-sheets, for the 6 mm ARC showed the eight spectral contributions consistent with that expected for casein (S3 Fig) (Table 1) [14,15]. No evidence of formaldehyde (methanediol or oligomers of methylene glycol, the known products of formaldehyde hydrolysis and subsequent condensation) or PDMS was observed, confirming the low concentrations present and localisation of these species to the top surface of the constrictor.

**Table 1:** Possible assignment of the characteristic Raman frequencies for the amide I band components of globular proteins found in the 6 mm ARC.

Raman shift / cm^-1^	Possible assignment
1602–1606	Aromatic sidechains
1615–1618	Aromatic sidechains
1631–1633	β-structure, low frequency component (weak)
1641–1646	H-O-H bending of bound water
1653–1658	Helical segments
1660–1663	Unspecified
1668–1675	β-structure, high frequency component (strong)
1680–1699	Turns

Analysis by ATR-FTIR spectroscopy (Table 2) supported the structural assignment of the constrictor as casein.

**Table 2:** Possible assignment of the characteristic infrared frequencies found in the ARCs.

Wavenumber / cm^-1^	Possible assignment
1050–1050	C-O bend
1230–1240	C-O stretch
1500	C-H bend
1500–1550	N-O stretch
1620–1640	C = C stretch
2840–3000	C-H stretch
3250–3300	N-H stretch

**Fig 4:**
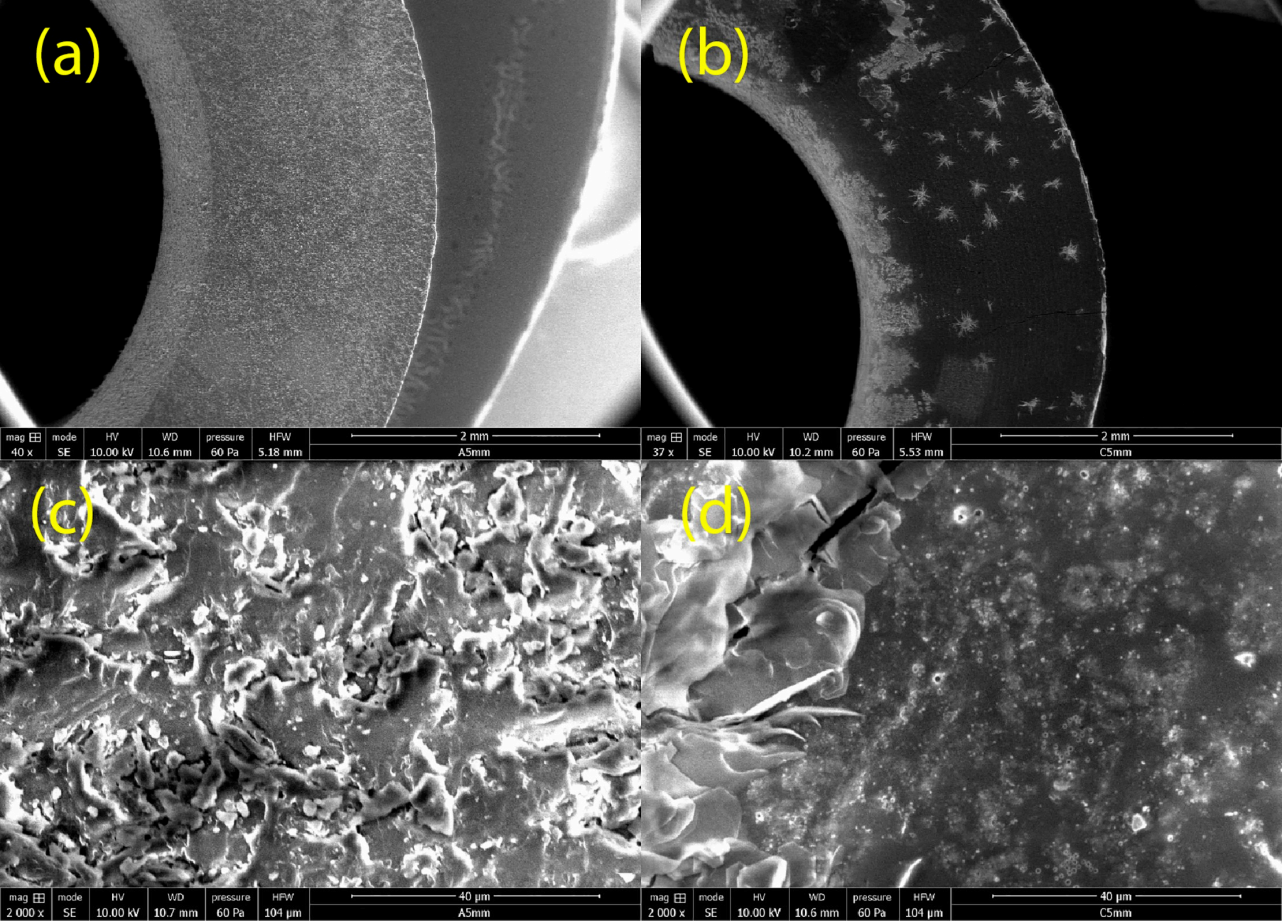
Scanning electron microscopic images of representative 5 mm ARC sections: (a) Native ARC at 40x magnification, (b) Post-Immersion ARC at 40x magnification, (c) Native ARC at 2000x magnification and (d) Post-Immersion ARC at 2000x magnification.

Electron microscopy analyses indicated that the topographical features of the ARC surfaces were consistent between all constrictors. SEM images (Fig 4) demonstrate the typical and very consistent features of the ARCs at both 40x and 2000x magnifications (at a working distance of ~10 mm). There was limited spatial heterogeneity across an individual surface seen on the macroscale. The circular ARC structure was very well defined with the majority of the top surfaces on a flat plane from the very outside edge to the centre of the ARCs. Close to the lumen each ARC also had a small region (~15–20% of surface area) of gradually downward sloping material. Equally when assessing the microscale, the surface morphologies remain relatively consistent (200x magnification and below). The surfaces were seen to be rough on the micronscale with ‘platelet’ topographical features and a rugged morphology.

### Post-immersion analysis

Following plasma immersion, there were notable changes in the composition of the ARCs as demonstrated by ToF-SIMS (Figs 1 and 2). Most noticeably, the depth profiles of the Ca^+^ ion (*m*/*z* = 39.95) showed significant trends in intensity with increasing sputtering time; the calcium content was seemingly depleted at the top surface, enriched at the sub-surface and then seen to sequentially decrease into the bulk of the constrictor. This was significantly different to the behaviour observed in the native ARC, where the Ca^+^ ion intensity showed a continual increase with analysis depth (Fig 2). Critically, aside from some slight anomalies, calcium intensities were lower overall in all post-immersion ARCs regardless of depth (except for sample 1 post-immersion). The apparent loss of calcium, specifically at the surface of the constrictors, could be attributed to the masking effect of PDMS. However, the sub-surface enrichment and overall depletion from the bulk was likely to be associated with the effect of calcium leaching induced during exposure to the plasma. This was supported by a general decrease in the Ca^+^ content throughout the entire ARC.

Compared to the native ARCs, a marked increase in the ion intensities of C_4_H_9_^+^, PO_2_^-^ and PO_3_^-^ was also observed in the plasma treated ARCs (Fig 1). This indicates an ingress of these ions into the bulk of the ARCs during plasma treatment. The elevation in CN^-^ ion intensity is specific to the ARC surfaces after plasma treatment (Fig 1) while bulk levels were equivocal. It is therefore difficult to determine whether this represents added material or, in addition to the calcium egress, reflects mobility and rearrangement of ARC bulk chemistry. The depth profiles for C_4_H_10_N^+^ (*m*/*z* = 72.10) and C_5_H_3_^-^ (*m*/*z* = 63.01) showed similar trends of universal elevation post-treatment; yet, the source of these elevations (i.e. directly from the plasma or as a consequence of changes to ARC structure induced during the plasma exposure) cannot be confirmed at this point.

**Fig 5:**
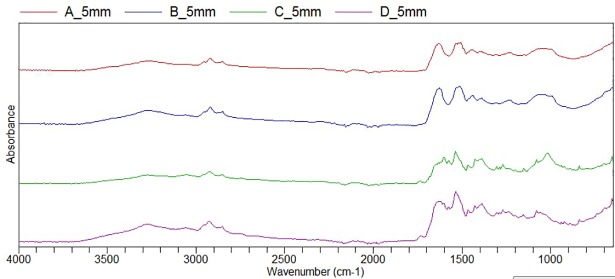
ATR-FTIR spectra of the 5 mm ARC in the Native state (A, B) and Post-Immersion (C, D) in canine plasma.

There were no significant changes in the Raman spectra recorded after exposure to canine plasma (Fig 3), indicating very little to no modification of the secondary structure of the proteins, with only a small decrease in the intensity of the peak at 1617 cm^-1^ in the spectra of the 3.5 and 5 mm ARCs (assigned as a stretching mode of an aromatic sidechain) noted. Subtle differences in the ATR-FTIR spectra (Fig 5), including increases in the intensity of the O-H stretching vibration in the range 3000–3100 cm^-1^, were additionally observed. Therefore, the results of vibrational spectroscopy, which both possess comparably large analysis volumes (on the microscale) and are thus inherently less surface sensitive than ToF-SIMS, indicate that the bulk structure remains largely unaffected by exposure to canine plasma.

Microscopic analysis further highlighted morphological changes to the ARCs after plasma immersion (Fig 4). SEM images of all ARCs showed that microscopic cracks in the surface developed. This was attributed to the hydration of the underlying material during immersion followed by drying on extraction. However, additional morphological changes were commonplace. These included an apparent adherence of extraneous material, most notably the presence of what appears to be crystal structures. These were scattered across the ARC surfaces but particularly frequent in the luminal region and were likely representative of salt deposition from the plasma. Aside from these features, on both the macro- and micro-scale the ARCs appears to have undergone smoothening, with far less rugged visual presentation. Again, this likely reflected the hydration of the casein during plasma exposure. Additionally, roughly circular aggregates of variable diameter (5–150 μm), were observed with variable periodicity on the ARC surface (Fig 4). The genesis of these is unclear, but may relate to surface focussed structural reorganisation of the sample.

### Plasma analysis

The plasma sample was analysed pre-immersion and had a total protein concentration of 4.77 g/dL, albumin concentration of 2.70 g/dL and globulin concentration of 2.07 g/dL. Post immersion aerobic and anaerobic cultures were both negative.

## Discussion

The results of our study prove that there is a high degree of intra- and inter-constrictor homogeneity in the native state. As demonstrated by Raman spectroscopy analysis of the ARCs, there are no significant variations in the secondary protein structure across the different constrictors or indeed from different areas within the same ARC. The same is largely true based on evidence from the other techniques, with all supporting the composition of all the ARCs as casein.

One slight variation noted during ToF-SIMS analysis of the native ARCs is the presence of PDMS on the surface of the ameroid constrictor. PDMS is a commonly used biomaterial and has a widely documented role in inflammation and thrombogenesis both *ex* and *in vivo*. [16,17] However, it is also a ubiquitous environmental contaminant and could have leached onto the constrictor surface either directly from the packaging or following removal from the packaging during handling. It is not known if it is present in sufficient concentration to result in an inflammatory response, but this does pose the question as to whether multiple handling events followed by re-sterilisation may increase the concentration of PDMS on the surface of the ARC, and thus whether multiple handling events may therefore affect the behaviour of the ARC when used *in vivo*.

Previous studies have hypothesised that the presence of formaldehyde may have a role as a trigger for perivascular inflammation and intravascular thrombosis. [7–9,18] One previous study has identified a possible formaldehyde signature ion CH_3_O^+^ at *m*/*z* 31, using ToF-SIMS analysis, [19] which was indeed detected in this study. Formaldehyde has a very simple, small and volatile chemistry (CH_2_O) and as such is often very hard to definitively identify, and due to the fragmentation process of ToF-SIMS other organic species may generate this ion. Therefore, the authors of the present study feel confident that the low intensity detected of a potential formaldehyde secondary ion signature, combined with the absence of its hydrolysis product (and subsequent condensation oligomeric forms) on the surface or sub-surface of the ARC would make it unlikely to be present in a sufficient concentration to trigger inflammation or thrombosis *in vivo*.

Following immersion in plasma there were no significant changes detected by Raman spectroscopy, demonstrating that plasma treatment does not significantly alter the secondary protein structure within the bulk of the ARC. Casein is known to have no disulphide linkages and therefore little tertiary structure. Subtle changes noted in the IR spectrum were consistent with that expected of exposure of a protein to plasma, largely assigned to an increase in the water content of the ARC post-immersion.

ToF-SIMS analysis demonstrated a significant change in the calcium profile, which is seemingly depleted in the near bulk, enriched near the surface and depleted again at the top surface. Moreover, the total amount of the Ca^+^ ion detected throughout the ARC is lower after plasma treatment. Calcium is, therefore, likely leached from within the ARC, concentrated near the surface and then washed away into the plasma at the absolute surface. Calcium is known to be important in determining the inter-protein interactions, with increasing concentration reducing the size of casein micelles but increasing aggregates seen. [20] Therefore, whilst the vibrational spectroscopy analysis suggested that the intra-protein structure was relatively unaffected by plasma exposure, it is probable that the macromolecular structure, driven by calcium-mediated inter-protein interactions, is. This is supported by the results of electron microscopy; it is clear that the surface topographical features were altered following plasma immersion, with increased number of artefacts seen surrounding the luminal surface.

A recent study utilizing a novel silicone-polyacrylic acid gradual venous occlusion device noted only minimal inflammation following 6 weeks implanted around the internal iliac vein of dogs, with no thrombus formation. [21] This is in contrast to the histological appearance of tissue following placement of an ARC and further suggests that the inflammatory reaction seen is in response to the ameroid material itself. Previous studies have shown that biomaterials thought to be inert and non-toxic can cause marked chronic inflammatory responses. [22] The trigger for this response has been indicted as a rapid and spontaneous adherence and adsorption of protein to the biomaterial surface. Factors that have been shown to affect the biocompatibility of a material when implanted in the body include surface free energy and wettability, surface chemistry and surface topography. [23,24] Possible sequelae of these issues of biocompatibility include protein adsorption, phagocytosis activation, platelet activation and clotting cascade activation, all of which have been noted in histological studies following implantation *in vivo*. [7–9,18] Our study has demonstrated variations that occur in both the surface chemistry and topography over the course of plasma immersion, which will affect the biomaterial-host interface. Although our study has failed to identify a compound responsible for an inflammatory trigger, it is likely that the change in the surface topography and chemistry affects the inherent biocompatibility of the ARC resulting in the changes observed histologically post-implantation.

Given the importance of the ARC surface for biocompatibility, the presence of PDMS contamination from surgically sterile manipulation raises the possibility that multiple handling events could potentially result in significant alteration of the ARC’s chemical surface, affecting how the biomaterial interface interacts *in vivo*. However, the concentration of PDMS or number of handling events required to result in this alteration in behaviour were not known or evaluated in the present study. Furthermore, although formaldehyde was detected within this study, it was at an extremely low level, and it is highly unlikely that this chemical bears any role in the inflammatory response seen *in vivo*.

## Supporting information

S1 Fig Mass spectra of the 5 mm ARC before and after plasma treatment showing the CH_3_O^+^ peak (*m*/*z* = 31.03) potentially associated with formaldehyde, throughout the depth profiling. (TIF)

S2 Fig CH_3_O^+^ ion (m/z = 31.03) profiles for ARCs: 3.5 mm, 5 mm, 6 mm and 7 mm. This shows there are variations between different constrictors and normalised intensity of CH_3_O^+^ is roughly between 1.4x10^-5^ and 1x10^-6^, which is closed to noise level (~5x10^-7^)
(TIF)

S3 Fig Comparison of the fitted experimental Raman spectra in the amide I region 1590–1720 cm^-1^ for the 6 mm ARC in the Native state (left, χ^2^ = 1.9) and Post-Immersion in plasma (right, χ^2^ = 2.8). The experimental spectra have been baseline corrected for residual fluorescence using a polynomial fitting model. The band shape of all eight individual fits is Gaussian. Direct comparison to the previously reported Raman spectra of casein is inherently challenging given differences in (i) the state of samples under analysis and (ii) the fitting models employed, the latter of which is compounded by relatively low signal to noise which ensures that the curve fitting procedures do not necessarily lead to unique solutions (the percentage abundance of the individual components was reported to vary by as much as 20% depending on the specific model). However, the ratio of α-helix to β-sheets of ~1:3 observed here is consistent with that noted previously for lyophilised casein and critically does not vary significantly between spectra taken from the Native and Post-Immersion samples. [15]
(TIF)

## Abbreviations

ARC: Ameroid ring constrictor
ATR-FTIR: attenuated total reflectance Fourier transform infrared
SEM: scanning electron microscopy
ToF-SIMS: Time-of-Flight Secondary Ion Mass Spectrometry
