# Supplementary figures and images for: Changes in chemical and ultrastructural composition of ameroid constrictors following in vitro expansion
Source: PLoS One. 2018 Nov 15;13(11):e0207471. doi: 10.1371/journal.pone.0207471 (PMC6237400; doi:10.1371/journal.pone.0207471)

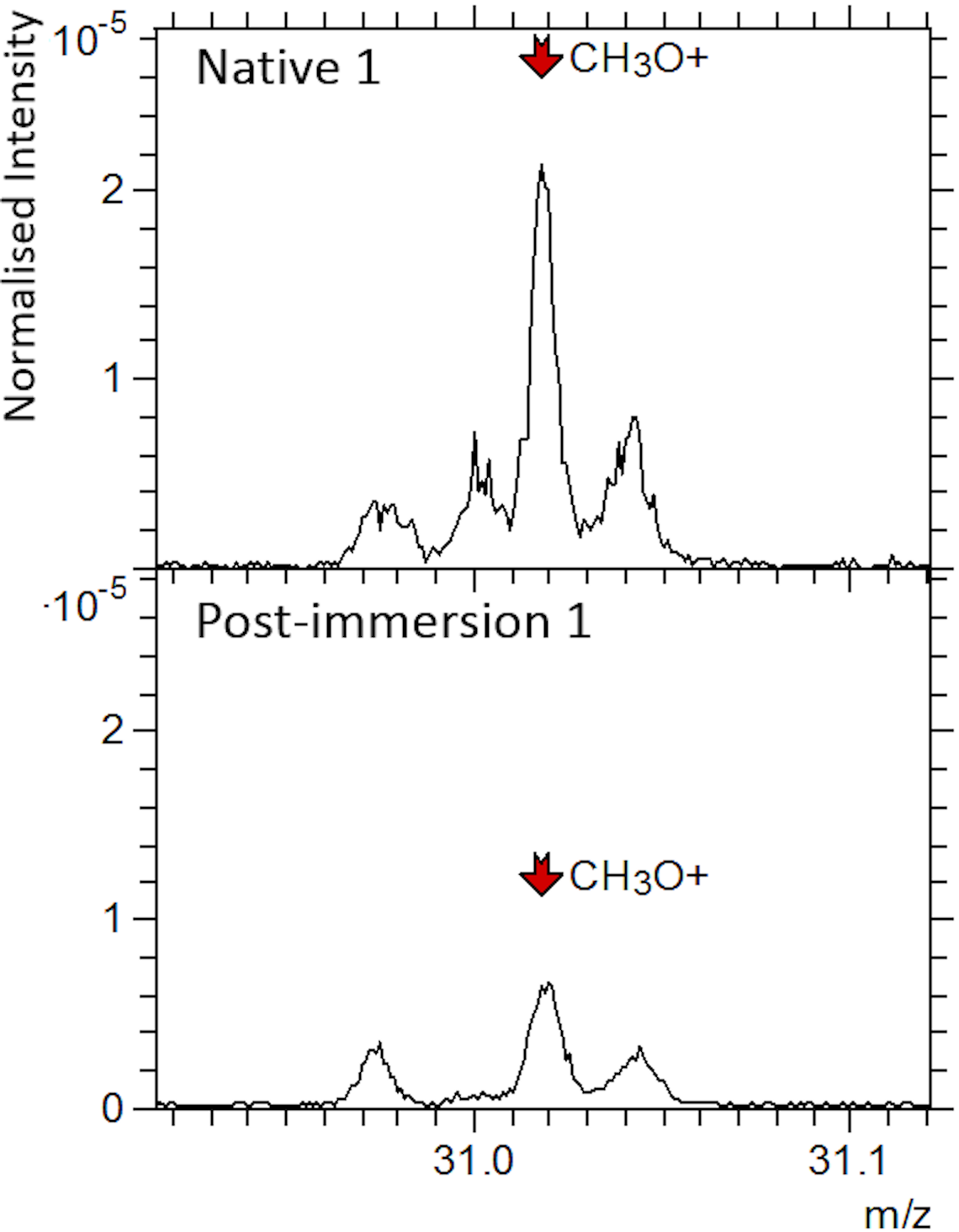

Supplement: S1 Fig — (TIF) [file pone.0207471.s001.tif]

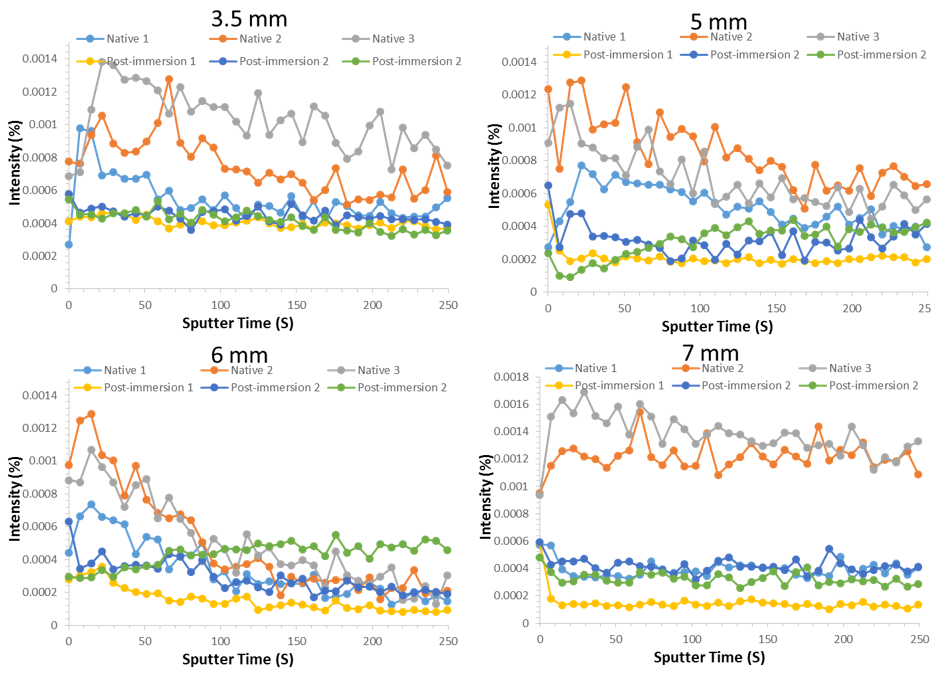

Supplement: S2 Fig — This shows there are variations between different constrictors and normalised intensity of CH3O+ is roughly between 1.4x10^-5^ and 1x10^-6^, which is closed to noise level (~5x10^-7^) (TIF) [file pone.0207471.s002.tif]

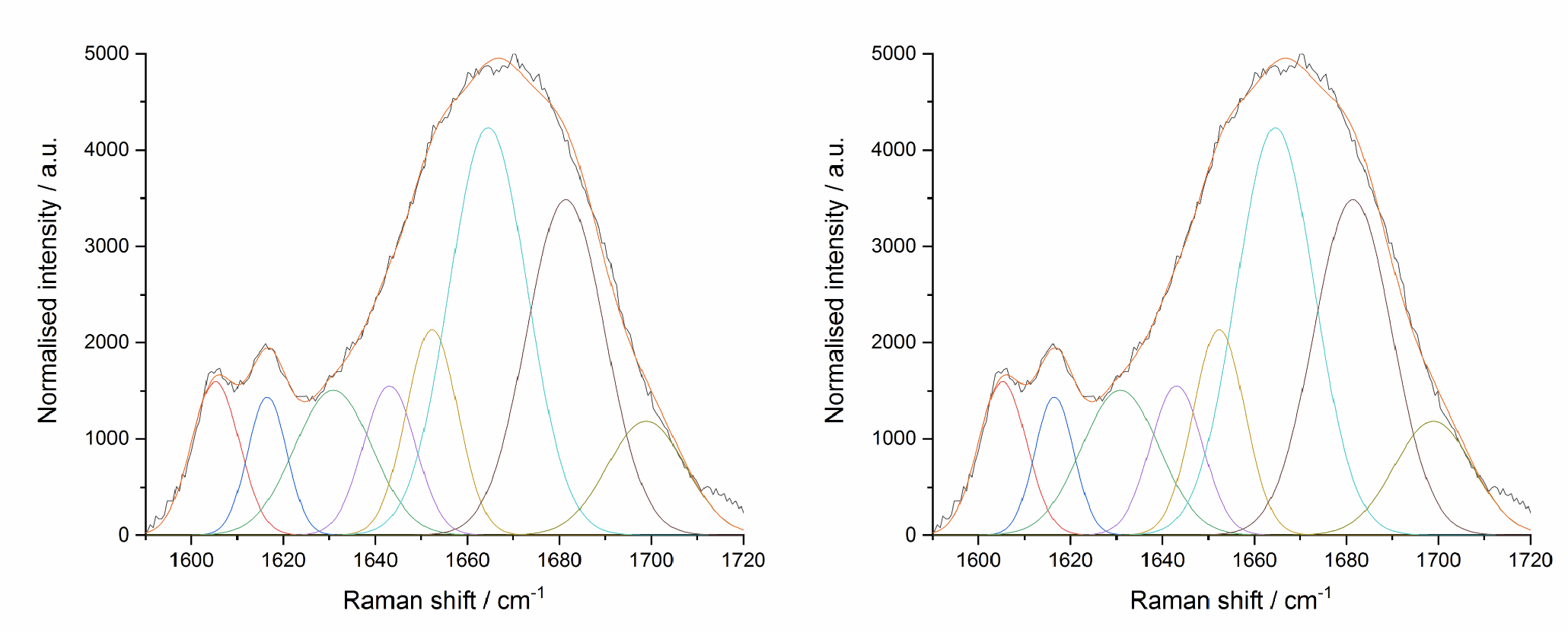

Supplement: S3 Fig — The experimental spectra have been baseline corrected for residual fluorescence using a polynomial fitting model. The band shape of all eight individual fits is Gaussian. Direct comparison to the previously reported Raman spectra of casein is inherently challenging given differences in (i) the state of samples under analysis and (ii) the fitting models employed, the latter of which is compounded by relatively low signal to noise which ensures that the curve fitting procedures do not necessarily lead to unique solutions (the percentage abundance of the individual components was reported to vary by as much as 20% depending on the specific model). However, the ratio of α-helix to β-sheets of ~1:3 observed here is consistent with that noted previously for lyophilised casein and critically does not vary significantly between spectra taken from the Native and Post-Immersion samples. [15] (TIF) [file pone.0207471.s003.tif]
